# Patterns of brain atrophy associated with episodic memory and semantic fluency decline in aging

**DOI:** 10.18632/aging.101186

**Published:** 2017-03-09

**Authors:** Amandine Pelletier, Charlotte Bernard, Bixente Dilharreguy, Catherine Helmer, Melanie Le Goff, Sandra Chanraud, Jean-François Dartigues, Michèle Allard, Hélène Amieva, Gwénaëlle Catheline

**Affiliations:** ^1^ University Bordeaux, ISPED, Centre INSERM U1219, F-33000 Bordeaux, France; ^2^ INSERM, ISPED, Centre INSERM U1219, F-33000 Bordeaux, France; ^3^ University Bordeaux, INCIA, UMR 5287, F-33000 Bordeaux, France; ^4^ CNRS, INCIA, UMR 5287, F-33000 Bordeaux, France; ^5^ EPHE, PSL Research University, F-33000 Bordeaux, France; ^6^ CHU de Bordeaux, F-33000 Bordeaux, France

**Keywords:** episodic memory decline, semantic fluency decline, grey matter atrophy

## Abstract

The cerebral substratum of age-related cognitive decline was evaluated in an elderly-cohort followed for 12 years (n=306). Participants, free of dementia, received neuropsychological assessments every two years and an MRI exam at baseline and four years later. Cognitive decline was evaluated on two broadly used tests to detect dementia: the Free and Cued Selective Reminding Test (FCSRT), a verbal episodic memory task, and the Isaacs Set Test (IST), a semantic fluency task. Using voxel-based approach, the relationship between cognitive decline with 1/ baseline grey matter volumes and 2/ grey matter volume loss between the two scans was explored. Baseline volumes analysis revealed that FCSRT and IST declines were both associated with lower volumes of the medial temporal region. Volumes loss analysis confirmed that both declines are related to medial temporal lobe atrophy and revealed that FCSRT decline was specifically associated with atrophy of the posterior cingulate cortex whereas IST decline was specifically related to temporal pole atrophy. These results suggest that cognitive decline across aging is firstly related to structural modifications of the medial temporal lobe, followed by an atrophy in the posterior midline structures for episodic memory and an atrophy of the temporal pole for semantic fluency.

## INTRODUCTION

Cognitive decline is commonly observed in normal aging [1–4]. A resurgence of interest on neuronal substrates of age-related memory decline in “cognitively normal” subjects is emerging since Alzheimer's Disease (AD) is now known to be preceded by a long presymptomatic stage. Indeed, memory deficit constitutes the hallmark of AD [5,6] and characterizes its preclinical stage [7]. Longitudinal population-based studies have revealed an insidious decline in episodic memory function several years to decades before the diagnosis of AD [8,9].

The progress in neuroimaging techniques opens an avenue for understanding *in vivo* brain mechanisms of age-related cognitive decline [2,3,10–13]. If some discrepancies still exist on the relationship between memory function and hippocampal volume when wide age-span is considered [14], most of volumetric grey matter studies conducted in elderly people report an association between volumes of medial temporal area or hippocampus (HC) and episodic memory performance [15–17]. Nevertheless, most past studies exploring the relationships between brain structure and function were based on cross-sectional MRI analyses (scans acquired at a single time point), which constitutes a limitation to address the atrophy and its co-occurrence with cognitive decline. Indeed, cross-sectional findings of smaller HC volume could reflect early-life variability rather than age-related change [18]. The concomitance of cognitive decline and brain tissue loss in healthy older population has been observed more recently thanks to MRI longitudinal studies allowing to track the within-person changes occurring over time in brain regions and the investigation of the two phenomena in the same temporal window. Nevertheless, most of these studies are focused on the Medial Temporal Lobe (MTL) [12,19–21] disregarding other regions potentially implicated in age-related memory decline such as frontal areas [16,22]. Finally, the longitudinal studies exploring the patterns of atrophy in the whole brain associated with age-related cognitive decline in a large population-based cohort are particularly scarce [23–25].

Hence, in this context, we compare the neuroanatomical substratum underlying age-related decline of performance in two standard neuropsychological tests widely used in clinical practice to detect dementia: the Free and Cued Selective Reminding Test (FCSRT; [26] used to assess episodic memory and the Isaacs Set Test (IST) [27] used to assess semantic fluency. The neuropsychological tests were administered in a sub-sample of the Three-City (3C) study (sample of Bordeaux), a large French population-based cohort monitored for 12 years, and MRI examinations were realized both at baseline and 4 years later. Whole-brain structural MRI analyses with no *a priori* hypothesis concerning the affected regions were conducted. These analyses should highlight similarities and differences in the topographical distribution of atrophy associated with declines of these widely used tests. First, we performed an analysis to investigate the association between grey matter (GM) volumes at baseline and decline of performance in the two memory tests. Secondly, a longitudinal MRI analysis was conducted to explore the concomitance of GM atrophy and weakening of performance in the two memory tests.

This two steps analysis should give some informations on spatio-temporal evolution of age-related volume loss. Indeed, we speculate that some regions should be highlited by both analyses reinforcing that physiopathological process is currently ongoing (under progress) and is then related to aging. For regions only highlited in the longitudinal analysis, we could speculate that they correspond to enlargement (extention) of the pathophysiological process. Finally, for regions only highlited in the cross sectional analysis, we could not distangle between age-related and previously-acquired characteristics.

## RESULTS

The mean age of participants was 72.7 years (SD=3.8, range=66.5-81.9). As may be seen in Table 1, 38.9% of the participants were men, 50.7% had a high level of education and 18.3% had one or two ApoE ε4 allele. At baseline, subjects presented a mean MMSE score of 28.1 (SD=1.7), a mean FCSRT free-recall score of 25.2 (SD=5.9), and a mean IST-60 score of 74.8 (SD=15.8). The mean decline for each memory test after 12 years of follow-up is presented in the Table 1. At baseline, the mean of GM volumes of the sample was of 480 cm^3^ (SD=41.3) and 4.13 years later (SD=0.3) the mean was of 473 cm^3^ (SD=42.8), which corresponds to an annual percent change in GM loss of 0.33% (SD=0.8).

**Table 1:** Characteristics of participants

n	306
**Demographic variables**	
Age (y) ±SD	72.7 ± 3.8
Gender: men	119 (38.9%)
Higher level of education	155 (50.7%)
ApoE ε4 (−/+) and (+/+)	56 (18.3%)
**Mean performance at baseline**	
MMSE ±SD	28.1 ± 1.7
IST 60 seconds ± SD	74.8 ±15.8
FCSRT free-recall ± SD	25.2 ± 5.9
FCSRT total-recall ± SD	45.0 ± 3.9
**Raw value of the decline slope**	
MMSE ± SD	−0.07 ± 0.1
IST 60 seconds ± SD	−1.06 ± 0.6
FCSRT free-recall ± SD	−0.15 ± 0.4
FCSRT total-recall ± SD	−0.12 ± 0.4

ApoE ε4 = the ε4 allele of the apolipoprotein E gene

MMSE = Mini-Mental State Examination (0-30 points; higher score indicates better cognitive status)

IST = Isaacs Set Test (higher score indicates better performance)

FCSRT = Free and Cued Selective Reminding Test (0-48 points; higher score indicates better performance)

### Baseline volumes analysis

**Figure 1:**
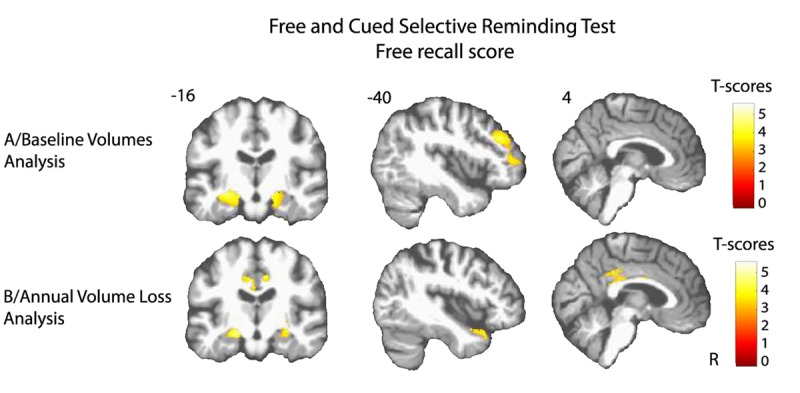
Cerebral substratum of episodic memory decline (**A**) Transversal VBM analysis: grey matter volumes at baseline and memory decline. (**B**) Longitudinal VBM analysis: grey matter annual rates of atrophy and memory decline. Each model was adjusted for age, sex, level of education, APOE4 allele carrier status and total intracranial volume (except for longitudinal VBM analysis). Clusters presenting statistically significant associations (q<0.05, topological cluster FDR corrected) with variance of the memory decline are overlaid on a spatially normalized TI image of one subject of the sample. Coordinates are given in millimeters the MNI space. R = right side.

**Figure 2:**
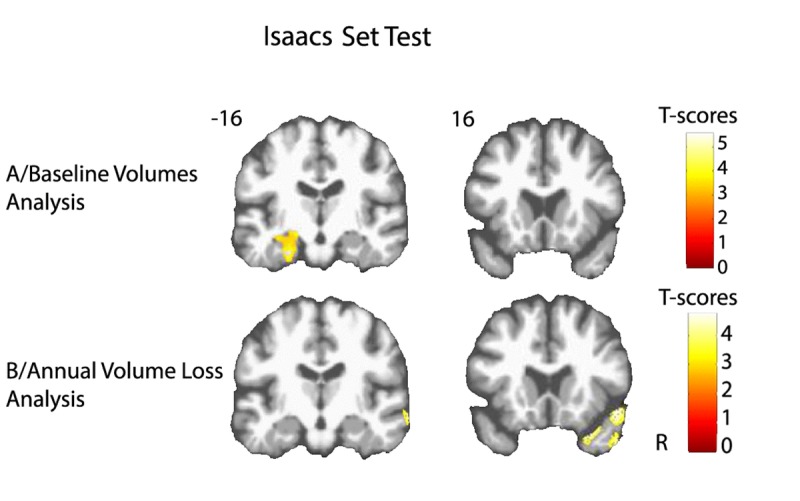
Cerebral substratum of semantic fluency decline (**A**) Transversal VBM analysis: grey matter volumes at baseline and fluency decline. (**B**) Longitudinal VBM analysis: grey matter annual rates of atrophy and fluency decline. Each model was adjusted for age, sex, level of education, APOE4 allele carrier status and total intracranial volume (except for longitudinal VBM analysis). Clusters presenting statistically significant associations (q<0.05, topological cluster FDR corrected) with variance of the fluency decline are overlaid on a spatially normalized TI image of one subject of the sample. Coordinates are given in millimeters in the MNI space. R = right side.

Lower volume of amygdala-hippocampus complex and parahippocampal regions at baseline were associated with greater decline of performance in both FCSRT free-recall (q<0.05, FDR corrected, cluster size k_E_=4519 voxels, Figure 1A) and IST tests (q<0.05, FDR corrected, cluster size k_E_= 6356 voxels, Figure 2A). Decline of FCSRT free-recall was also related to lower volumes in left middle and inferior frontal cortices (Figure 1A). Analysis with baseline MRI did not reveal any significant association with the decline of FCSRT total-recall. Whatever the decline considered (FCSRT free-recall, FCSRT total-recall or IST), no significant association was observed with the reverse contrast (higher decline related to higher GM volumes).

### Annual volume loss analysis

**Figure 3:**
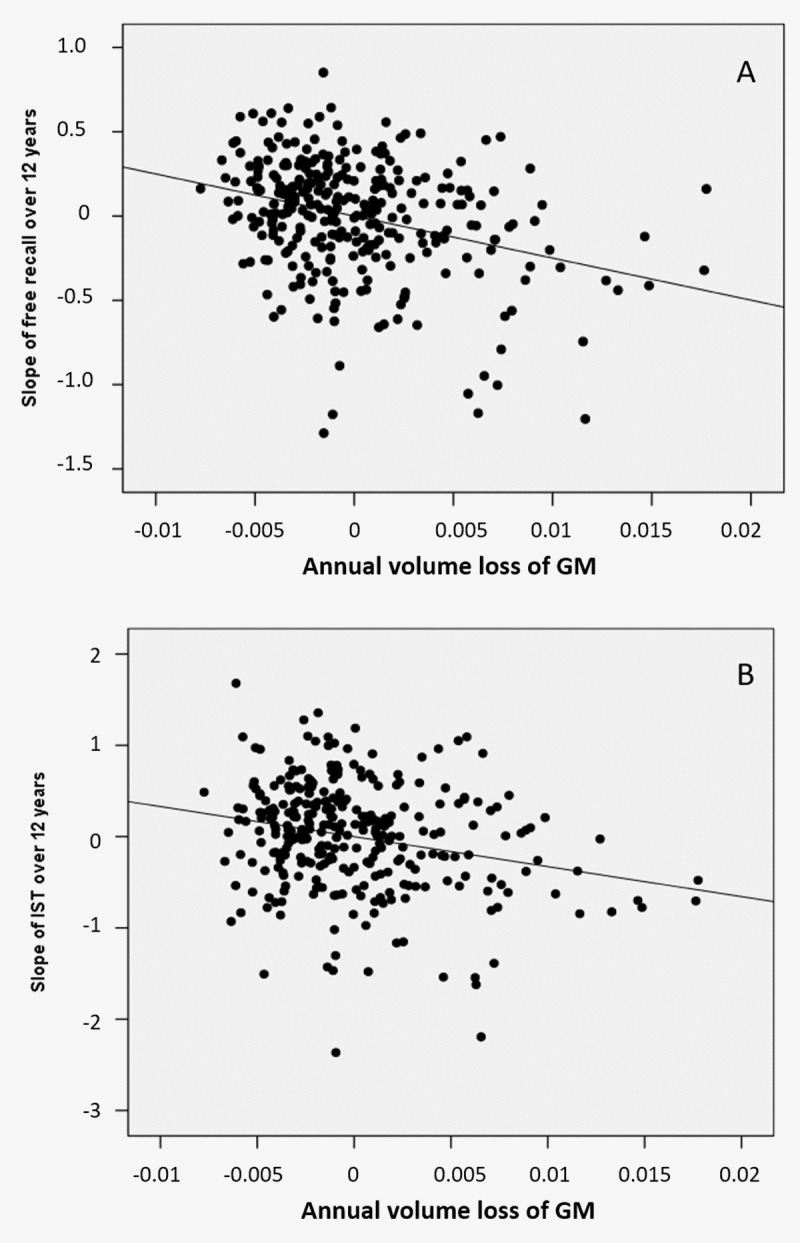
Partial regression plots between annual volumes loss at the level of the mesio-temporal cluster and cognitive decline (**A**) of the FCSRT free-recall (**B**) of the IST in models including age, gender, level of education and ApoE genotype as covariables.

Atrophy of bilateral amygdala-hippocampus complex and parahippocampal region was associated with the decline of the FCSRT free-recall (q<0.05, FDR corrected, cluster size k_E_= 2313 voxels, Figure 1B and Figure 3A). The same cerebral regions were associated to the decline in total-recall (data not shown). Free-recall decline was also associated with atrophy of bilateral posterior cingulate cortex (PCC)/precuneus region and of the left temporal pole (Figure 1B). Regarding IST, the analysis showed that decline in performance was associated with atrophy of the right temporal and fusiform cortices, the temporal pole, and the parahippocampal region (q<0.05, FDR corrected, cluster size k_E_ = 2330 voxels, Figure 2B), and to a less significant threshold of the hippocampal region (p<0.001, uncorrected, cluster size k_E_ = 100 voxels, Figure 3B). Finally, no significant association was observed with the reverse contrast (higher decline related to lower GM atrophy).

## DISCUSSION

This study provides evidence for neuroanatomical changes associated with age-related decline in two memory tests, one involving episodic memory and one involving semantic fluency. We first observed that declines of episodic memory and semantic fluency are not associated with widely distributed GM modifications across the brain but rather to a restricted number of cerebral regions. Moreover, weakening of performance in both tests is associated with reduced volumes of the MTL. Finally, longitudinal analysis revealed a specific association between atrophy of the PCC/precuneus for episodic memory decline and of the temporal pole for semantic fluency decline.

### Episodic memory decline

According to our results, age-related atrophy of the MTL contributes to episodic memory change in healthy elderly subjects. Indeed, decline of FCSRT performance was concomitant to atrophy of the left anterior part of the MTL, including the hippocampus and parahippocampal cortex. Our results are in full accordance with recent longitudinal ROI studies based on automatic segmentation of the hippocampus [12,21,24] and cortical thickness measurement [23,24].

Interestingly, our whole brain analysis also reveals that episodic memory decline is related to atrophy of the PCC/precuneus. In the longitudinal MRI study conducted by Fjell and collaborators, while the PCC/precuneus regions presented a pronounced age-related atrophy, the results failed to show the contribution of such atrophy to episodic memory decline [25]. Based on a large sample, our study probably benefited from higher statistical power (132 versus 306 healthy elderly participants). Indeed, our findings are in accordance with functional MRI studies showing that the posteromedial cortex plays a crucial role in episodic memory processes [28–31]. More precisely, age-related episodic memory decline has been associated with a failure of deactivation of this posteromedial region [32], and with a decreased intrinsic connectivity between the PCC and the HC [33]. More recently, an impaired modulation ability of the posteromedial cortex was related to increasing age, greater amyloid burden and worse episodic memory performance [34].

The FCSRT free-recall score is clearly associated to MTL atrophy in our non-demented subjects whereas FCSRT total-recall is not, which is in accordance with previous study showing that the sum of free and cued recall (termed total recall) has high discriminative validity for dementia, wheareas free recall is useful to predict the development of future dementia [35].

### Semantic fluency decline

As for episodic memory scores, the cross-sectional analysis highlighted that decline in semantic fluency is related to lower volumes in amygdala-hippocampal and parahippocampal regions. Although the MTL is widely considered as the neuroanatomical substrate of episodic memory [36,37], neuroimaging studies have extended its implication in semantic memory [38–40]. Some authors suggested that the involvement of left hippocampus in semantic information processing result from use of strategies relying on autobiographic processes and episodic recollections optimizing semantic memory functioning [39,41–43]. Recent studies have highlighted a selective recruitment of the hippocampus in verbal fluency tasks, particularly in semantic ones [38,39,44–46].

The longitudinal analysis revealed that the decrease in semantic fluency performance was associated with temporal pole atrophy. Our findings are in line with numerous studies evidencing the crucial role of the anterior temporal lobe in the semantic representation of words, objects, people, and social concepts [47–54]. Several investigations have shown that semantic deficit arose from damage to lateral and anterior regions of the temporal lobes, rather than to medial regions [37,55–57].

### Distinct and common patterns of atrophy

Taking together, the results gathered from MRI cross-sectional and MRI longitudinal analyses provide some clues about the spatio-temporal dynamics of cerebral atrophy associated to age-related memory decline. Indeed, association with the MTL was observed in cross-sectional and longitudinal analyses for both memory tests, suggesting that atrophy of the MTL in aging is the earliest and most significant morphological change underlying decline for these two tests in normal aging. One could have expected to observe an association of both tests with frontal cortex alterations as numerous studies have underlined its implication in age-related cognitive decline, leading to postulate “the frontal aging hypothesis” [22,58–60]. According to our findings, it is not the specific atrophy of frontal regions which better explains the weakening of episodic memory and semantic fluency performance. Nonetheless, it is important to underline that our study relying on morphological measures does not address the question of the functional implication of frontal region in cognitive performance. Therefore, the lack of results on GM morphological measures does not exclude age-related functional impairment or anatomical disconnection of the frontal lobe [61–63].

Our findings suggest that episodic memory decline is firstly related to atrophy in hippocampal region, and secondly to atrophy in posterior midline structures. This spatio-temporal dynamics of atrophy is in accordance with studies demonstrating PCC atrophy in advanced stages of memory impairment like patients with mild cognitive impairment or AD [64]. Similarly, it appears from our results that age-related semantic fluency decline is firstly associated with hippocampal atrophy and secondly with temporal pole atrophy.

### Methodological considerations

The major strength of this study is the combination of a large population-based cohort study with both baseline and 4-year follow-up MRI data which provided a detailed description of the spatial progression of atrophy associated with age-related changes in two tests, classically used to detect dementia among elderly. Moreover, since the participants were prospectively followed during 12 years, the memory trajectories were obtained over more than one decade in this population. Even though some authors suggest that non-linear models may provide an accurate characterization of cognitive trajectories [65], we used a linear mixed model to compute the slopes of cognitive decline to stay consistent with MRI analyses for which we have only two time points. Further studies with more MRI time points will be necessary to investigate non-linear effects of aging process [66] related to non-linear cognitive decline. In a supplementary analysis, we compared the sub-sample with one MRI (n=357) of the whole baseline cohort to the sub-sample with two MRI used in our analysis. We observed that subjects included in our analysis are youngers and slighty preserved from hippocampal atrophy compared to the others indicating a classical non-random attrition, weakening the generalization of our result to the population.

### Conclusion

In a large sample of 306 healthy elderly participants, the present study shows the implication of MTL atrophy in the weakening of both episodic memory and semantic fluency performance in normal aging. In addition to this common substratum, the longitudinal analysis of MRI data also evidences atrophy in specific regions associated with these two tests. Indeed, in addition to hippocampal atrophy, episodic memory decline is also associated with precuneus/PCC atrophy whereas semantic fluency decline is specifically related to temporal pole atrophy.

## METHODS

### Participants

The participants are gathered from Bordeaux subset of the 3C study, a longitudinal multicenter population-based cohort initially designed to evaluate risk factors of dementia. Subjects were non-institutionalized individuals aged ≥ 65 years and randomly recruited from electoral lists. Details on the study have been described previously [67]. The study protocol was approved by the ethics committee of Kremlin-Bicêtre University Hospital (Paris, France), and all participants provided written informed consent. Since the 1999-2000 baseline inclusion, neuropsychological assessments were administered by trained psychologists at each follow-up visit occurring at 2, 4, 7, 10 and 12 years. Global cognitive efficiency was evaluated with the Mini-Mental State Evaluation (MMSE) [68]. Verbal episodic memory was evaluated with the FCSRT. The FCSRT consists in learning 16 words referring to 16 semantic categories. First, for the encoding phase, four cards, each displaying four target items are presented to the participant. After a semantic category cue is given, the participant is required to name the item corresponding to each category. After each card presentation, the subject has to recall immediately the four items. The second phase consists in three successive recall trials, separated by an interference task. Each trial includes a free recall and a cued recall for the missed items. Finally, the last phase consists in a delayed recall of the 16 word-list including a free and a cued recall. For our analyses, we used the following FCSRT subscores: the total score of the three free recall trials (free-recall) and the total score of the three free and cued recalls (total-recall). The IST measures semantic fluency. Subjects have to name as many words as possible belonging to a semantic category in 60 seconds. Four categories are successively used: colors, animals, fruits and cities. For this study, the score used was the sum of words provided across all 4 categories. Longitudinally, the participant slopes for FCSRT and IST scores were estimated using a linear mixed model with random intercepts and slopes, which allows the best linear estimations of decline slopes taking into account all the points of assessment (from baseline to 12-year follow-up). So, the more the slope is negative, the more the decline is important.

At each follow-up, subjects suspected of dementia were seen at home by a neurologist or a geriatrician who confirmed the diagnosis and specified the etiology of dementia. Following this assessment, a definitive diagnosis was made by a panel of independent neurologists to obtain a consensus, according to the Diagnostic and Statistical Manual of Mental Disorders - 4th edition criteria and the National Institute of Neurological and Communicative Disorders and Stroke-Alzheimer's Disease and Related Disorders Association criteria for AD [69].

**Figure 4:**
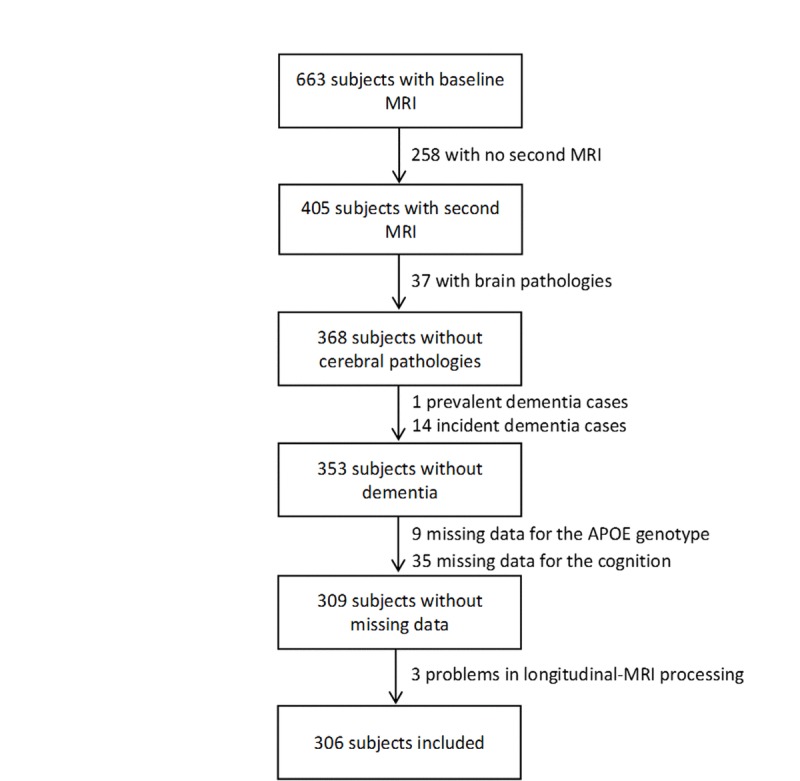
Flowchart of participants selection for the analyses

In Bordeaux, 663 MRI examinations were performed at baseline. Among them, 405 subjects underwent a second MRI examination 4 years later. Subjects were excluded from the analyses if they presented severe brain pathologies (n=37, tumor, stroke, severe leucoaeriosis), a prevalent dementia (n=1), an incident dementia between the two scan examinations (n=14), missing data for the ε4 allele of the apolipoprotein E gene (ApoE ε4) (n=9), for the FCSRT and the IST scores (n=35), and a technical problem in longitudinal MRI processing (n=3). Finally, MRI analyses were conducted on 306 subjects (Figure 4).

### MRI acquisition

MRI examination was performed using a 1.5 Tesla Gyroscan Intera system (Philips Medical Systems, Netherlands) equipped with a quadrature head coil. The same scanner and the same sequence were used for the baseline and the follow-up MRI. Anatomical high resolution MRI volumes were acquired in transverse plane using a 3D MPRAGE T1 weighted sequence (TR/TE 8.5/3.9 ms, 10-degree flip angle, matrix size 256×256, FOV 240 mm, yielding 124 slices and slices thickness of 1 mm, voxel size 0.94×0.94×1 mm^3^). Head motions were minimized by use of tightly padded clamps attached to the head coil.

### Baseline-MRI processing

An optimized Voxel-Based Morphometry (VBM) procedure [70] implemented in the VBM8 toolbox (revision 343, http://dbm.neuro.uni-jena.de/vbm) of Statistical Parametric Mapping 8 (SPM8) (Welcome Laboratory of the Department of Cognitive Neurology, Institute of Neurology, London, UK, http:www.fil.ion.ucl.ac.uk./spm) was used with default parameters to analyze brain volumes. Briefly, images were denoised, segmented into grey matter (GM), white matter (WM) and cerebro-spinal fluid (CSF) maps, warped to the Montreal Neurological Institute (MNI) space with a DARTEL type non-linear registration and modulated to preserve volume information. Finally, they were smoothed with an isotropic 8-mm Full Width at Half Maximum (FWHM) gaussian filter. All segmented partitions were visually checked by an experienced operator (CB) so as to discard poor quality processes. Total intracranial volume (TIV) was calculated by adding global GM, WM and CSF volumes after masking out the cerebellum because the acquisition for some MRI scans did not cover the whole cerebellum.

### Longitudinal-MRI processing

A longitudinal VBM procedure implemented in the VBM8 toolbox was used to analyze atrophy, i.e. volume loss between the two MRI scans. For processing, default parameters were used. For each subject, the second image was registered to the baseline image and a mean image was created which served as reference for a second realignment. Then, the realigned images were bias-corrected using the mean image as a reference and were segmented. Furthermore, using the segmentation of the mean image, the spatial normalization parameters were estimated using DARTEL. Those estimated parameters were applied to normalize the segmented images. Finally, an ultimate realignment of those normalized images was performed. All segmented partitions were visually checked by an experienced operator (CB) so as to discard poor quality processes. Maps of the GM annual volume loss were then calculated using the difference between the baseline and follow-up normalized GM maps and using the delay in years between the two MRI scans:

$$\frac{\text{Baseline normalized GM maps}-\text{Follow-up normalized GM maps}}{\text{Baseline MRI Time}-\text{Follow-up MRI Time}}$$

Annual volume losses of GM maps were finally smoothed at 8-mm FWHM. So, the higher GM rate is, the greater atrophy is.

### Statistical analyses

Firstly, we explored the relationship between GM morphology and episodic memory and semantic fluency declines using SPM8 multiple linear regressions. Secondly, we explored the relationship between GM annual volume loss and episodic memory and semantic fluency declines using SPM8 multiple linear regressions. For each analysis, decline slopes for FCSRT or IST were used as predictors.

The analyses were systematically run with age, gender, level of education, ApoE genotype and TIV (except for longitudinal-VBM analysis) as co-variables. For each analysis, positive and negative contrasts were assessed. Results were presented corrected for multiple comparisons using the cluster topological false discovery rate (FDRc; SPM8) and were considered as significant for a statistical threshold of q<0.05 for clusters using a cluster height threshold of p<0.001 and an extent threshold calculated under Gaussian Random Field Theory [71].
