# Correction: Patterns of brain atrophy associated with episodic memory and semantic fluency decline in aging

**DOI:** 10.18632/aging.101241

**Published:** 2017-05-16

**Authors:** Amandine Pelletier, Charlotte Bernard, Bixente Dilharreguy, Catherine Helmer, Melanie Le Goff, Sandra Chanraud, Jean-François Dartigues, Michèle Allard, Hélène Amieva, Gwénaëlle Catheline

**Aging (Albany NY). 2017; 9: 741–752**. PMCID: PMC 5391228 PMID: 28278492

In this article, the last and first name of corresponding author is mixed up: http://www.aging-us.com/article/101186/text

The correct citation of this author is: **Gwénaëlle Catheline.**

